# Variant Aldehyde Dehydrogenase 2 (ALDH2*2) as a Risk Factor for Mechanical LA Substrate Formation and Atrial Fibrillation with Modest Alcohol Consumption in Ethnic Asians

**DOI:** 10.3390/biom11111559

**Published:** 2021-10-21

**Authors:** Chung-Lieh Hung, Kuo-Tzu Sung, Shun-Chuan Chang, Yen-Yu Liu, Jen-Yuan Kuo, Wen-Hung Huang, Cheng-Huang Su, Chuan-Chuan Liu, Shin-Yi Tsai, Chia-Yuan Liu, An-Sheng Lee, Szu-Hua Pan, Shih-Wei Wang, Charles Jia-Yin Hou, Ta-Chuan Hung, Hung-I Yeh

**Affiliations:** 1 Department of Medicine, Mackay Medical College, New Taipei City 25245, Taiwan; jotaro3791@gmail.com (C.-L.H.); 8905012@gmail.com (K.-T.S.); zhang@mmc.edu.tw (S.-C.C.); yenyu1012@gmail.com (Y.-Y.L.); jykuo5813@gmail.com (J.-Y.K.); chsu007@gmail.com (C.-H.S.); stsai22@jhu.edu (S.-Y.T.); henry76@mmh.org.tw (C.-Y.L.); anshenglee33@gmail.com (A.-S.L.); shihwei@mmc.edu.tw (S.-W.W.); jiayin@mmh.org.tw (C.J.-Y.H.); hung0787@ms67.hinet.net (T.-C.H.); 2 Division of Cardiology, Departments of Internal Medicine, MacKay Memorial Hospital, Taipei 10449, Taiwan; 5819.5819@mmh.org.tw; 3 Institute of Biomedical Sciences, Mackay Medical College, New Taipei City 25245, Taiwan; 4 Department of Critical Care Medicine, MacKay Memorial Hospital, Taipei 10449, Taiwan; 5 Department of Physiology Examination, MacKay Memorial Hospital, New Taipei City 25160, Taiwan; carrie@ms1.mmh.org.tw; 6 Department of Health Policy and Management, Johns Hopkins University Bloomberg School of Public Health, Baltimore, MD 21205, USA; 7 Division of Gastroenterology, Department of Internal Medicine, MacKay Memorial Hospital, Taipei 10449, Taiwan; 8 Graduate Institute of Medical Genomics and Proteomics, College of Medicine, National Taiwan University, Taipei 10051, Taiwan; shpan@ntu.edu.tw; 9 Genome and Systems Biology Degree Program, Academia Sinica and National Taiwan University, Taipei 10617, Taiwan; 10 Doctoral Degree Program of Translational Medicine, College of Medicine, National Taiwan University, Taipei 10051, Taiwan; 11 Mackay Junior College of Medicine, Nursing and Management, Taipei 11260, Taiwan

**Keywords:** aldehyde dehydrogenase 2 (ALDH2), alcohol, aldehyde, 4-hydroxynonenal (4-HNE), peak atrial longitudinal strain (PALS), strain rates, atrial fibrillation (AF)

## Abstract

Aldehyde dehydrogenase 2 (ALDH2) rs671 polymorphism is a common genetic variant in Asians that is responsible for defective toxic aldehyde and lipid peroxidation metabolism after alcohol consumption. The extent to which low alcohol consumption may cause atrial substrates to trigger atrial fibrillation (AF) development in users with ALDH2 variants remains to be determined. We prospectively enrolled 249 ethnic Asians, including 56 non-drinkers and 193 habitual drinkers (135 (70%) as ALDH2 wild-type: GG, rs671; 58 (30%) as ALDH2 variants: G/A or A/A, rs671). Novel left atrial (LA) mechanical substrates with dynamic characteristics were assessed using a speckle-tracking algorithm and correlated to daily alcohol consumption and ALDH2 genotypes. Despite modest and comparable alcohol consumption by the habitual alcohol users (14.3 [8.3~28.6] and 12.3 [6.3~30.7] g/day for those without and with ALDH2 polymorphism, *p* = 0.31), there was a substantial and graded increase in the 4-HNE adduct and prolonged PR, and a reduction in novel LA mechanical parameters (including peak atrial longitudinal strain (PALS) and phasic strain rates (reservoir, conduit, and booster pump functions), *p* < 0.05), rather than an LA emptying fraction (LAEF) or LA volume index across non-drinkers, and in habitual drinkers without and with ALDH2 polymorphism (all *p* < 0.05). The presence of ALDH2 polymorphism worsened the association between increasing daily alcohol dose and LAEF, PALS, and phasic reservoir and booster functions (all P*_interaction_*: <0.05). Binge drinking superimposed on regular alcohol use exclusively further worsened LA booster pump function compared to regular drinking without binge use (1.66 ± 0.57 vs. 1.97 ± 0.56 1/s, *p* = 0.001). Impaired LA booster function further independently helped to predict AF after consideration of the CHARGE-AF score (adjusted 1.68 (95% CI: 1.06–2.67), *p* = 0.028, per 1 z-score increment). Habitual modest alcohol consumption led to mechanical LA substrate formation in an ethnic Asian population, which was more pronounced in subjects harboring ALDH2 variants. Impaired LA booster functions may serve as a useful predictor of AF in such populations.

## 1. Introduction

Aldehyde dehydrogenase 2 (ALDH2) is a mitochondrial enzyme that catalyzes the oxidation of acetaldehyde, which is the main toxic product of ethanol metabolism that causes major organ damage after alcohol consumption [1,2]. As a common genetic variant, the aldehyde dehydrogenase 2 glu504lys polymorphism (rs671) is prevalent in 35–57% of East Asian regions and is responsible for deficient ALDH2 enzyme activity and a lower threshold of tissue damage that is attributable to alcohol consumption [2,3]. ALDH2 is also known to catalyze aldehyde oxidation as a secondary product of lipid peroxidation (e.g., 4-hydroxy−2-nonenal (4-HNE)) and confers protection against oxidative stress through reactive oxygen species (ROS) reduction beyond the effect of ethanol detoxification [4,5]. Restoration of ALDH2 deficiency can reportedly reverse the myocardial damage (e.g., fibrosis) caused by acetaldehyde toxicity that arises due to excessive alcohol consumption and was shown to reverse the extent of cardiac remodeling and mechanical dysfunction [2,6].

A great body of literature has demonstrated that even moderate doses of alcohol consumption increase the risk of atrial fibrillation (AF) [7,8,9]. It was also proposed that abstinence from alcohol consumption may reduce the recurrence of arrhythmias in regular consumers with atrial fibrillation [10]. These findings highlight the toxic effects of prolonged alcohol consumption on the atrial tissue. Not only the quantity but also the patterns of alcohol consumption (e.g., regular alcohol consumption versus binge drinking) or genetic factors may affect the relationship between alcohol consumption and cardiac dysfunction [11]. While previous epidemiological studies demonstrated a higher AF incidence in modest alcohol consumers, the relationship between low-dose alcohol consumption and AF incidence remains controversial in women and men with an alcohol consumption rate of <14 drinks/week and <21 drinks/week, respectively [7,8,12]. Previously, we demonstrated that mild-to-moderate alcohol consumption might be associated with sub-clinical left atrial (LA) mechanical dysfunction in a large population-based study reported in ethnic Asians [13]. We also showed that ALDH2 polymorphism might exacerbate left-ventricular (LV) systolic dysfunction in subjects that are prone to habitual drinking by conducting a study involving a community-based cohort [14], which further highlighted the role of ALDH2 polymorphism in alcohol-induced subclinical cardiomyopathy.

Thus far, the extent to which alcohol consumption may predispose LA to abnormal electromechanical substrate formation as a clinical marker for AF incidence, especially in patients harboring ALDH2 polymorphism in certain Asian regions, remains unclear. Therefore, we investigated the role of ALDH2 polymorphisms with regard to several LA mechanical indices in regular, modest alcohol consumers.

## 2. Materials and Methods

### 2.1. Study Subjects

We prospectively recruited 268 study participants from our outpatient clinics to investigate the association between aldehyde dehydrogenase 2 (ALDH2) SNP rs671 and left atrial (LA) electromechanical functions (from May 2014 to May 2016) in a population of individuals presenting with habitual alcohol consumption. Aldehyde dehydrogenase 2 (ALDH2) SNP rs671 genotype data were available for 260 study participants as either homozygous (A/A, rs671) or heterozygous (G/A, rs671) information. Our previous study reported a mean of 32.5% and an SD of 8.0% on the left atrial (LA) deformational measure (peak atrial longitudinal strain (PALS)) in the light-to-moderate alcohol consumption group (≥1 drink/week) and a mean of 37.7% and SD of 8.0% in the non-drinker group, which could result in an effect size (Cohen’s *d*) of 0.40. Based on this information, we designed a study to enroll study participants presenting with habitual alcohol consumption versus non-habitual alcohol consumers in a prespecified 3:1 allocation ratio, leading to a sample size of nearly 180:60, with a power of 95% and an α rate of 5%. We collected baseline demographic information, including anthropometrics, 12-lead body surface electrocardiogram (ECG), medical history (including hypertension, type 2 diabetes, treatment for hyperlipidemia, coronary artery disease (CAD), and cerebrovascular events), and medication usage. Biochemical data regarding the fasting glucose level, liver enzymes, lipid profiles, blood urea nitrogen, and creatinine levels were obtained using the Hitachi 7170 Chemistry Analyzer (Hitachi Corp., Hitachinaka, Ibaraki, Japan). Renal function was expressed as the eGFR using the MDRD formula. Prevalent heart failure (HF) and urgent medical conditions, such as acute coronary events, were excluded. Informed consent was obtained from all study participants.

Daily alcohol intake was estimated using a structured questionnaire, where different types of alcoholic beverages, amounts of alcohol consumed, and frequency of use were self-reported. Briefly, study participants were requested to provide information about the amount, frequency, and duration of their consumption of beer, liquor, wine, and strong wine, as well as the exact amount of alcohol consumed on a certain occasion. Total weekly alcohol consumption for each participant was calculated in grams of pure alcohol by multiplying the frequency of each consumed alcoholic beverage, the exact dose of ethanol derived from the amount, ethanol concentration (alcohol by volume, e.g., beer: 3.5–4.5%), and the specific gravity of ethanol (0.79 g/mL) in each type of alcohol beverage. The questionnaire used for the exact quantification of alcoholic beverage consumption in the present study was validated in our previously published report [13]. Binge-drinking behavior was defined as the consumption of five or more drinks for men or four or more drinks for women in the short term [15].

**Figure 1:**
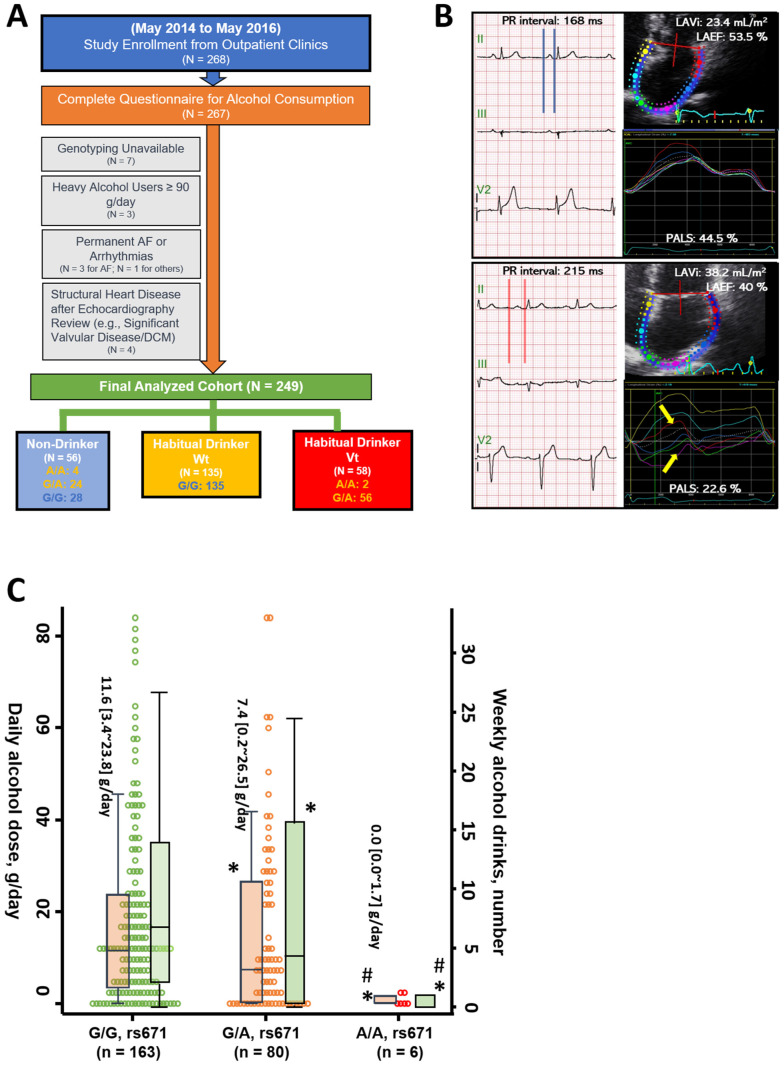
(**A**) Schematic representation of the setting and exclusion criteria of the samples that were included in the present study. (**B**) Illustration of mechanical LA substrates that were used in the present study. A 44-year-old male without a history of regular alcohol consumption (upper panel) demonstrating a normal PR interval with a small LA size and preserved PALS; a 40-year-old male, ALDH2 polymorphism carrier with a history of chronic excessive alcohol consumption (>20 gday), showing a prolonged PR interval accompanied by LA remodeling and impaired PALS. (**C**) Distribution and daily alcohol intake (gday, median level, and IQR) across distinct ALDH2 genotypes as G/G, G/A, and A/A, rs671 (3 groups). One drink is equal to 12 gpure alcohol. Orange bars represent daily alcohol dose (g and green bars represent weekly alcohol drinks, respectively. Both were presented as median and IQR. * *p* < 0.05 vs. ALDH2 wild type (G/G), ^#^ *p* < 0.05 vs. ALDH2 polymorphism (G/A) via ANOVA.

This study passed the Institutional Review Board of MacKay Memorial Hospital, Taipei (14MMHIS069). All study participants provided informed consent. The study flowchart of the eligibility and exclusion criteria is detailed in Figure 1A.

### 2.2. Conventional Cardiac Structural and Diastolic Functional Assessment

Our echocardiography instrument was equipped with a 2–4 MHz transducer (M4S or 3S-RS) in the screening program using the Vivid 7/i system (GE Healthcare, Little Chalfont, United Kingdom). The standard echocardiography imaging protocol for conventional cardiac structures included a linear assessment for the LV internal end-diastolic diameter, wall thickness (septal and posterior wall), and derived LV mass index (American Society of Echocardiography criteria). The LA volumes were measured using the biplane Simpson method with and without the indexed to body surface area (BSA) as the LA volume index (LAVi). The LA emptying fraction (LAEF) was calculated as follows: 100 × (maximal LA volume-minimal LA volume)/maximal LA volume. The mitral inflow E/A ratio was determined via mitral inflow pulsed-wave Doppler of early (E) and late diastolic (A) filling velocities at the tip of the mitral leaflets from the apical 4-chamber view. Data on the TDI-based mitral annulus relaxation velocities (e’) were acquired and averaged from both the lateral and septal mitral annulus positions with high frame rates, with LV filling pressure estimated as the E/e’ (average) ratio.

### 2.3. Assessment of the PR Interval Using Body Surface Electrocardiography (ECG)

Study participants underwent routine body surface ECG examinations, during which, recordings were obtained using autonomic instruments (Page Writer Trim III; Philips, Andover, MA, USA). The PR interval was determined with initiation at the T-P junction at the start point of the P-wave to the initiation of the QRS segment. Measurements of the PR interval were conducted by using lead II with a 0.1 mm calibration, as in the published protocol [16] (Figure 1B).

### 2.4. Assessment of Phasic LA Functional Imaging Using Novel LA Deformational Markers

Cardiac images of three continuous cardiac cycles from any view were acquired at a rate of 60–100 frames per second for each study participant. Speckle-tracking analyses for LA function were performed using commercial software and algorithms (EchoPAC, version 10.8, GE Vingmed Ultrasound, Norway). For obtaining good-quality phasic LA deformational measures, image acquisition was carefully optimized for the LV apical 4-chamber and 2-chamber views to avoid foreshortening. Peak atrial longitudinal strain (PALS) and phasic strain rate measures (reservoir, conduit, and booster pump functions, respectively) (Figure S1) were generated for each atrial segment from LV apical 2- and 4-chamber views using offline analysis, according to a previously published protocol [17]. Since our quantitative LA deformational algorithm was based on a regular heart cycle and rhythm to better delineate the dynamic LA curves, subjects with irregular rhythm during the image acquisition (e.g., atrial fibrillation (AF), frequent VPCs, or AV block) were further excluded from the LA strain/strain rate analysis (Figure 1A). Data on the representative LA deformational indices in each study participant were then derived from the mean of both LV apical 2- and 4-chamber data and are presented as absolute values |x| for statistical convenience. The rater for the LA deformational measures was blinded to the clinical information.

### 2.5. CHARGE-AF Score Calculation

To assess whether the use of novel LA deformational indices might outperform clinical risk factors for incident AF, we further calculated the CHARGE-AF as a composite clinical risk score in the present study [18]. Briefly, the CHARGE-AF score was calculated using the following formula:

CHARGE-AF risk score = 0.508 × age (5 years) + 0.248 × height (10 cm) + 0.115 × weight (15 kg) + 0.197 × systolic blood pressure (20 mmHg) − 0.101 × diastolic blood pressure (10 mmHg) + 0.359 × current smoker + 0.349 × antihypertensive medication + 0.237 × diabetes + 0.701 × congestive heart failure + 0.496 × myocardial infarction. In the present study, antihypertensive medication was defined as a medical history of hypertension because all hypertension subjects were administered with antihypertensive medications. Congestive heart failure was not present in this study.

### 2.6. Assessment of Circulating Aldehyde By-Product 4-Hydroxynonenal (4-HNE)

The aldehyde by-product 4-hydroxynonenal (4-HNE) is considered to be a measure of oxidative damage based on a lipid peroxidation assay that is analyzed using ELISA. The level of HNE adducts was determined via an enzyme immunoassay using the OxiSelect™ HNE-adduct competitive ELISA kit using serum from venipuncture sampling according to the manufacturer’s instructions; the estimation was performed from the absorbance measurement using the Sunrise Microplate Absorbance Reader (Tecan, Switzerland). The HNE-adduct sampling for each participant was obtained at the baseline visit when the structured questionnaire for alcohol use was filled and was available in 245 (98.4%) study participants.

### 2.7. Definitions of Prevalent AF

Baseline prevalent AF diagnosis information was extracted from the electronic medical chart view (as paroxysmal, persistent, or permanent) according to contemporary clinical practice guidelines [19], periodic routine ECG surveys (biannually), and symptom-driven 24 h Holter studies. We also conducted a follow-up of the study participants during the subsequent five years using the same definition as the baseline. The presence of AF was defined as positive if either baseline or follow-up AF was present (as a binary outcome variable).

### 2.8. Statistical Analysis

Continuous data were presented as mean ± standard deviation (SD) (if normally distributed) or median with IQR (25th–75th) (if not normally distributed), with categorical data expressed as numbers and percentages. The unpaired Student’s *t*-test or Mann-Whitney U test was used to analyze the differences in continuous variables between non-alcohol consumers and habitual alcohol consumers, when appropriate; one-way ANOVA was used to test the differences in continuous data among the three categorization groups (for example, alcohol non-users, alcohol users without ALDH2 polymorphism, and alcohol users with an ALDH polymorphism with a post hoc Bonferroni correction) for paired comparisons. Categorical data were compared using the chi-square or Fisher’s exact test, as appropriate. Normality was evaluated using the Kolmogorov-Smirnov test. Linear regression analysis was performed to explore the associations between daily alcohol consumption (per 1 gincrement/day) and a variety of LA mechanical parameters. We further determined whether the presence of ALDH2 polymorphism modified the association between alcohol consumption dose and LA mechanical parameters with several clinical covariates serving as clinical confounders. To examine whether modest alcohol consumption harboring ALDH2 polymorphism was associated with a higher prevalence of AF (baseline or during follow-up conducted for 5 years) compared to non-drinkers or regular drinkers without ALDH2 polymorphism, we further examined the individual odds ratio using ordinal logistic regression analysis (by considering the ordered categorical variables (non-drinkers and habitual drinkers without and with ALDH2 polymorphism) as a predictor; non-drinkers were considered to be the reference). The model was further subjected to adjustments for the CHARGE-AF score. By transforming all the LA mechanical parameters into z-scores (standard scores), we also examined their independent contributions to prevalent AF individually after adjustment for daily alcohol consumption and the presence of ALDH2 polymorphism (binary) using a logistic regression model and after sequential adjustment for the CHARGE-AF score.

## 3. Results

### 3.1. Baseline Demographics

Habitual drinkers (n = 193) presented with a significantly lower percentage of genotypic ALDH2 polymorphism (G/A or A/A, rs671) than non-drinkers (n = 56) (30.1 vs. 50%, *p* = 0.006). Habitual drinkers were less likely to be female, presented with comparable age, higher systolic blood pressure, had marginally higher diastolic blood pressure and CHARGE-AF score (10.8 ± 1.2 vs. 10.5 ± 1.1, *p* = 0.048), and were more likely to exhibit binge-drinking behavior (total n = 62, 31.1 vs. 3.6%, *p* < 0.001) when compared to non-drinkers (Table 1). The median alcohol intake was 14.1 g/day (IQR: 7.6~28.6 g/day) and the median alcohol consumption duration was 16.0 (5.0~30.0) years for habitual drinkers, although daily alcohol use was comparable in habitual drinkers stratified by considering ALDH2 polymorphism (14.3 [8.3~28.6] and 12.3 [6.3~30.7] g/day for those without and with ALDH2 polymorphism, *p* = 0.31 for a post hoc pairwise comparison between two groups). For all study participants, a significant and graded reduction of daily alcohol use was observed in subjects harboring ALDH2 polymorphism with a surprisingly low level of alcohol intake in ALDH2 homozygous subjects (G/A, rs671: 7.7 [0.2~28.6] g/day and A/A, rs671: 0.0 [0.0~1.7] g/day) compared to those without ALDH2 polymorphism (11.8 [3.4~24.9] g/day) (all paired *p* < 0.05) (Figure 1C). In addition, alcohol consumption duration was slightly longer in habitual drinkers without ALDH2 polymorphism compared to those carrying ALDH2 polymorphism (median: 20.0 [5.0~30.0] vs. 15.0 [5.0~30.0] years for those without and with ALDH2 polymorphism, *p* = 0.25 for post hoc pairwise comparison between two groups), though this did not reach statistical significance. The 4-HNE level was significantly higher in habitual drinkers and was the highest in drinkers with ALDH2 polymorphism (34.2 ± 7.7 vs. 36.6 ± 7.1 and 43.3 ± 12.5 μg/mL, respectively, *p* < 0.001). Habitual drinkers, regardless of the presence of ALDH2 polymorphism, were generally more likely to be active smokers or prior smokers, more likely to have a history of hypertension, hyperlipidemia treatment, and a higher prevalence of CAD and CVD compared to non-drinkers (*p* < 0.05). Overall, the biochemical data were broadly comparable between the groups.

Higher 4-HNE levels were inversely associated with PALS (r = −0.26, *p* < 0.001), LA reservoir (r = −0.27, *p* < 0.001), and conduit function (r = −0.18, *p* = 0.003), and showed a borderline correlation with booster pump function (r = −0.12, *p* = 0.066). These relationships remained significant after further adjustment for age and sex (including the booster function, all *p* < 0.05). Marginal associations were observed between the 4-HNE level, LV wall thickness (r = 0.13, *p* = 0.05), PR interval (r = 0.10, *p* = 0.10), increased LAVi (r = 0.10, *p* = 0.10), and lower LAEF (r = −0.10, *p* = 0.11).

### 3.2. Association of ALDH2 Polymorphism with Novel Functional LA Indices in Modest Alcohol Consumers

Habitual drinkers were significantly associated with greater wall thickness, along with a borderline increased LV mass index (Table 2). Measures of LV geometry were broadly comparable in habitual drinkers, irrespective of the presence of ALDH2 variants. Data regarding LA electromechanical properties and novel functional parameters showed a significantly longer PR interval, lower PALS, and worsened LA strain rate parameters (including LA reservoir, conduit, and booster pump functions) in habitual drinkers compared with non-drinkers (*p* < 0.05). LAVi and LAEF were not significantly different between drinkers and non-drinkers. In general, a longer duration of alcohol consumption (per decade increment) was associated with worse PALS and reservoir and booster pump function (adjustment for age and sex) (Figure S2).

Despite the overall comparable LV geometric measures, regular drinkers with ALDH2 polymorphism presented with a longer PR interval, along with substantially worsened novel LA functional parameters across non-drinkers and habitual drinkers without and with ALDH2 polymorphism, except for LAEF (all *p* < 0.05, ANOVA) (Table 2). LAVi was marginally higher in regular alcohol consumers with ALDH2 polymorphism than that in non-drinkers (*p* = 0.078) and regular drinkers without ALDH2 polymorphism (*p* = 0.054). An adjusted regression using non-drinkers and habitual drinkers without and with ALDH2 polymorphism considered as an ordinal category showed a significantly longer PR interval and worsened novel LA functional strain/strain rates; however, it was not observed with respect to LAEF in drinkers versus non-drinkers (*p* < 0.05) (Table 3).

**Figure 2:**
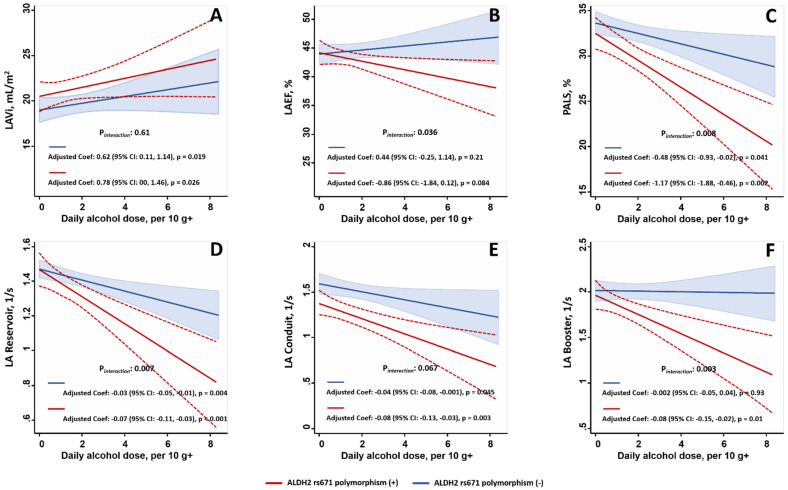
Linear regression models that were used in the present study, which demonstrate that ALDH2 polymorphism modified the association between daily alcohol intake (g/day) and worsened LA mechanical parameters (including LAVi (**A**), LAEF (**B**) and novel LA deformation markers (**C**–**F**) with steeper slopes for LAEF, PALS, and LA strain rates of the reservoir and booster pump functions (P*_interaction_*: <0.05). The models were subjected to adjustment for age, body mass index, renal function in terms of eGFR (as continuous variables), gender, medical histories of hypertension, diabetes, coronary artery disease, medications used for hyperlipidemia, and active smoking status (in contrast to non-smokers plus prior smokers) (all as binary variables). Dashed lines refer to 95% confidence interval.

**Figure 3:**
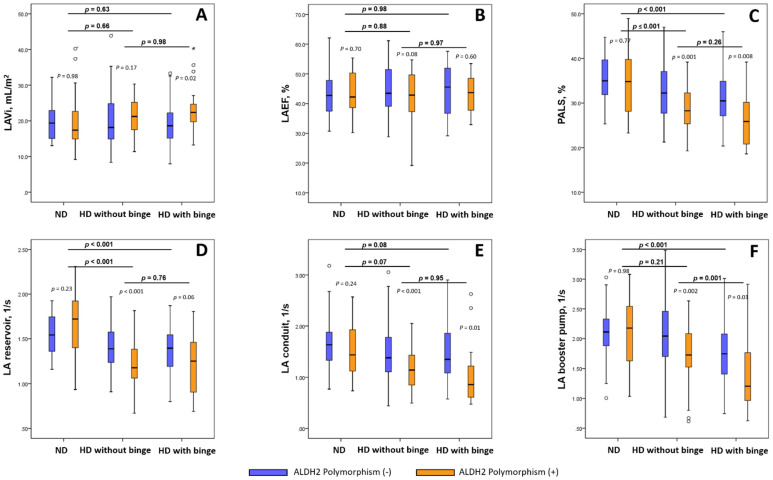
Comparisons of novel LA mechanical parameters with regard to the presence of ALDH2 polymorphism in subgroups stratified across non-drinkers (n = 56) and habitual drinkers presenting without (n = 132) and with (n = 61) binge-drinking behavior. Comparisons of worsened LA mechanical parameters (including LAVi (**A**), LAEF (**B**) and nov-el LA deformation markers (**C**–**F**) between ALDH2 polymorphism (−) and (+) two groups within any subcategory. * refers to statistical significance (*p* < 0.05) between ALDH2 polymorphism (−) and (+).

The use of linear regression models demonstrated that the presence of ALDH2 polymorphism resulted in steeper linear relationships between the daily alcohol consumption dose and lower LAEF (P_interaction_: 0.04), worsened PALS (P_interaction_: 0.008), and increased deterioration in the LA reservoir and booster pump functions (P_interaction_: 0.006 and 0.003, respectively) (Figure 2). Multivariate linear regression models that were used to explore the associations between clinical confounders (including daily alcohol intake), ALDH2 polymorphism, and a variety of LA mechanical parameters are shown in Table S1. Broadly, the presence of ALDH2 polymorphism worsened the novel LA mechanical parameters but did not worsen the LAEF in habitual drinkers without (n = 132) or with (n = 61) binge-drinking behavior; however, these differences were not observed in non-drinkers (Figure 3). Binge drinking superimposed on habitual alcohol consumption exclusively impaired LA booster pump function but did not affect other LA mechanical indices compared to the finding that was reported for habitual consumption alone (1.66 ± 0.57 vs. 1.97 ± 0.56 1/s, *p* = 0.001).

### 3.3. Association of ALDH2 Polymorphism with Prevalent AF in Modest Alcohol Consumers

**Figure 4:**
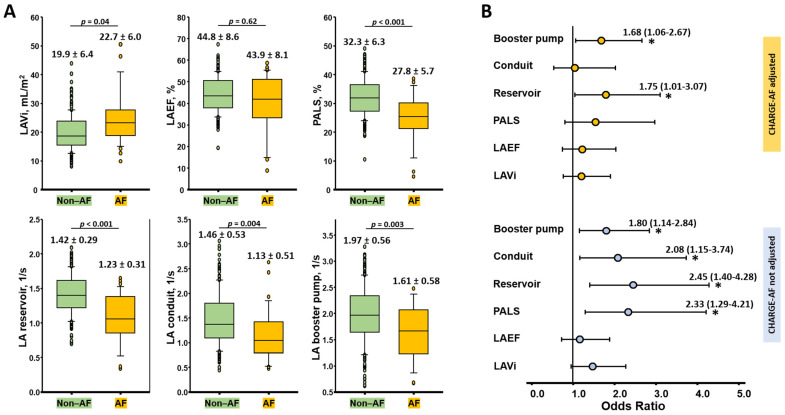
(**A**) Comparisons of various LA mechanical parameters between subjects with AF (n = 25) and those without AF (n = 224). (**B**) Odds ratios for the different LA mechanical parameters that were used for identifying AF. The baseline model for each LA parameter as a predictor was transformed into a standardized z-score and was separately examined. All parameters were subjected to a uniform adjustment for daily drinking dose and presence of ALDH2 polymorphism (binary variable) (lower panel). Multivariate models were further subjected to adjustment for CHARGE-AF scores (upper panel). PR (OR: 1.10, 95% CI: 0.72–1.69, *p* = 0.65), LAVi (OR: 1.47, 95% CI: 0.95–2.27, *p* = 0.086), and LAEF (OR: 1.16, 95% CI: 0.72–1.88, *p* = 0.54) were non-significant predictors prior to the adjustment for CHARGE-AF. * denotes *p* < 0.05 in the models.

In total, 25 (10.0%) study participants presented with paroxysmal/persistent AF (at baseline or during follow-up) in the present study after the exclusion of those with baseline permanent AF (n = 3) during echocardiography. Among the three individuals that presented with permanent AF excluded at baseline, two demonstrated ALDH2 polymorphism. Among those showing AF, the prevalence of ALDH2 Vt was comparable to the non-AF counterparts (40.0 vs. 33.9%, *p* = 0.55) with significantly higher daily alcohol consumption (14.3 [8.6~28.6] gday vs. 10.0 [1.7~23.0] gday, *p* = 0.016). Those who had AF demonstrated a slightly larger LAVi (22.7 ± 1.2 vs. 19.9 ± 6.4 mL/m^2^, *p* = 0.04), a non-significant difference in LAEF (43.9 ± 8.1 vs. 44.8 ± 8.6%, *p* = 0.62), and significantly lower PALS and all strain rate parameters (all *p* < 0.05) (Figure 4A). Although the PR interval was numerically longer in subjects with AF compared to the non-AF counterparts, the difference was not statistically significant (167.1 ± 26.8 vs. 163.6 ± 23.4 ms, *p* = 0.48).

A higher prevalence of AF was observed in modest alcohol users that harbored ALDH2 polymorphism compared to regular drinkers without ALDH2 polymorphism and non-drinkers (15.5 vs. 11.1 and 1.8%, respectively; *p* = 0.042) and the values remained relatively unchanged after adjustments were performed for baseline CHARGE-AF scores (Table 4); however, the underlying risk factors and baseline demographics were comparable between the two habitual alcohol consumption groups with and without ALDH2 polymorphism (Table 1). After considering the daily alcohol consumption and the presence of ALDH2 polymorphism, the phasic LA parameters of the reservoir and booster pump functions (adjusted OR: 1.76 (95% CI: 1.01–3.07), *p* = 0.046 and 1.68 (95% CI: 1.06–2.67), *p* = 0.028 per 1 standardized unit increment) remained independent indicators in AF after the CHARGE-AF score adjustments (Figure 4B). The PR interval did not show an independent association (OR: 1.10, *p* = 0.65) with AF after the adjustments were performed for alcohol intake and ALDH2 polymorphism.

## 4. Discussion

In the present study, we examined the impact of ALDH2 polymorphism on a variety of LA electromechanical changes in an ethnic Asian population with individuals exhibiting modest daily alcohol consumption compared to non-drinkers. Even with modest alcohol consumption, habitual alcohol drinkers consistently showed substantial sub-clinical LA mechanical impairment prior to overt structural remodeling, which was particularly prominent in those harboring ALDH2 polymorphism, which acted as a key acetaldehyde catalyzer after alcohol consumption. Our study is the first to explore these relationships, with phasic LA mechanical characteristics considered as potential discriminators, and to investigate the use of these novel markers for the identification of AF in such populations.

### 4.1. Association of Patterns of Alcohol Consumption with Atrial Substrate Vulnerability and AF

AF was well documented as a clinical disease following the “holiday heart syndrome” that was documented with binge drinking [20]. Acute alcohol consumption leads to the exhibition of a concentration-dependent pro-arrhythmic effect, which is primarily due to the occurrence of acute changes in LA electromechanical properties [15,21,22]. As a distinction from acute alcohol consumption, chronic alcohol exposure may also promote LA substrate formation, resulting in AF initiation or maintenance [23]. Regular low levels of alcohol consumption (e.g., ≥2 drinks/day) were associated with increased AF incidence (approximately 30% higher) as a threshold effect that was reported in large population-based studies and a meta-analysis [7,8,9,12,24,25]. Electrophysiological disturbances (such as dysregulated vagal tone, hyper-adrenergic status, or prolonged intra-atrial conduction time) [7,8,9,12,26] due to chronic alcohol intake were proposed as key pathophysiologies underlying AF development. In addition to electrophysiological effects, mechanical LA substrate formation, which is characterized by structural remodeling and suppressive mechanical properties [23,27], can occur with chronic alcohol consumption and is mainly driven by elicited oxidative stress (e.g., reactive oxygen species (ROS)) [28,29], cytotoxic aldehyde accumulation owing to lipid peroxidation (e.g., acrolein and HNE protein adducts), myofibrillar protein loss, and myofilament Ca^2+^ mishandling [30,31]. Despite a reported non-linear relationship between low level (>12 gday) alcohol consumption and AF incidence [9], thus far, uncertainty and controversy regarding the threshold of mechanical substrate formation with smaller quantities (1 drink/day) of regular alcohol consumption related to new-onset AF remain. Morphological LA remodeling or dilation is reportedly an intermediate phenotype for AF and is deemed to be a substrate marker for atrial arrhythmias/AF with chronic alcohol intake [23,32]. Compared to the 4-HNE adduct, which was identified as a circulating biomarker with cyclic turnover, we assumed that an LA mechanical assessment could better reflect the true burden and chronicity of myocardial atrial damage that occurs due to long-term alcohol ingestion [33]. As per the findings reported in our previously published study, as well as those published by Voskoboinik et al., it was demonstrated that mechanical LA dysfunction may gradually occur at a low and moderate dose [13,34] (~1 drink/day), as evidenced by using novel sensitive markers of echocardiography-based myocardial strain or via cardiac MRI. The postulated mechanisms by which mechanical LA substrate formation with chronic alcohol consumption may trigger atrial arrhythmias that are attributable to accumulative oxidative damage and fibrotic replacement (e.g., upregulated TGF-β1/collagen) within atrial myofibers, which may interfere with electrical conduction, promoting and perpetuating AF [23,35].

### 4.2. ALDH2 Polymorphism Modifies the Association of Alcohol Use with LA Mechanical Substrate Vulnerability

ALDH2, which is a key enzyme that is involved in alcohol detoxification, is well known as the key enzyme that is involved in the clearance of toxic acetaldehyde accumulation that arises from ethanol consumption and in the metabolism events of lipid peroxidation (for example, 4-HNE) [2,36]. *ALDH2* polymorphism has a low prevalence in Western society but the prevalence can be as high as 52% in certain Asian regions [37]. ALDH2 deficiency owing to the decreased *ALDH2* enzyme activities arising from the existence of a dysfunctional A allele of the *ALDH2* SNP that is commonly present in Asians (e.g., G/A or A/A, rs671) can lead to reduced capacity in the conversion of acetaldehyde to acetic acid [36,38] and reduced catalysis of oxidation of aldehyde derived from alcohol consumption. Since ALDH2 was also shown to confer protection against oxidative stress [39], individuals harboring dysfunctional *ALDH2* SNPs are also more susceptible to cardiovascular damage due to ROS-induced stress, especially in hypoxic conditions, leading to LA substrate formation and AF initiation and maintenance [40]. In fact, ROS-induced TGF-β1 overexpression and atrial fibrosis can be reversed via supplementation with an ALDH2-selective activator (Alda-1). Restoration of ALDH2 deficiency was shown to ameliorate 4-HNE accumulation with AF reduction in experimental animal models [35]. Our previous study in a community-based cohort showed impaired sub-clinical LV mechanics in relation to mild-to-moderate alcohol consumption, and this was closely influenced by genetic components (ADH, ALDH2 polymorphism). Although contemporary AF risk-scoring systems incorporated lifestyle factors, such as smoking behavior and alcohol consumption, as potential predictors, genetic susceptibility (e.g., ALDH2 polymorphism) in these systems remain largely underused and not well calibrated for ethnic Asians [18]. Genetic components were shown to play a role in AF risk prediction, and the association between *ALDH2* SNPs, alcohol consumption, and AF incidence remains largely unexplored [41] by large-scale epidemiological surveys. Concordant with previous reports with an extension of our previous findings, we showed that daily alcohol consumption as low as >1 drink per day accompanied by ALDH2 polymorphism showed significant LA strain reduction, supporting the concept of high susceptibility to LA damage with low ethanol exposure in ethnic Asians. While a previous study showed that a higher AF risk was observed for frequent drinking patterns rather than binge-drinking behavior [42], our data showed that binge-drinking behavior likely further impacted mechanical LA booster pump function when superimposed on chronic alcohol consumption. We found that impaired LA booster function could be considered to ascertain subjects harboring ALDH2 polymorphism with modest alcohol consumption and could be used to independently identify AF in these subjects. As an active and key modulator against the presence of impaired LV diastolic kinetics, the LA booster pump function emerged as a strong marker that can be used for identifying AF incidence [43,44]; however, the exact mechanisms and clinical significance of our findings warrant further research.

### 4.3. Novel LA Mechanical Substrate as a Surrogate Marker for AF Vulnerability

We found that LA functional disturbance, assessed using phasic deformational measures, was independent of the CHARGE-AF score used for identifying AF in an ethnic Asian population when exposed to modest alcohol consumption. CHARGE-AF was used as a composite clinical risk score; however, data on alcohol consumption, genetic profiles, and LA substrate information were not incorporated into the scoring system [18]. Therefore, our present work highlights the potential applicability of novel LA surrogate markers in ethnic Asian subjects harboring the ALDH2 rs671 genetic polymorphism, especially in those individuals with modest alcohol consumption. The utilization of LA deformational measures may provide an opportunity to delineate these functional disturbances in habitual alcohol consumers at an earlier stage, highlighting further implications in the clinical setting. Therefore, this may exert a considerable impact on AF prevention in ethnic Asian regions and may likely lead to a change in our daily practice guidelines for AF prevention and intervention. Herein, we propose the utility of an LA functional marker as a surrogate for imaging LA substrates for high-risk subjects harboring the ALDH2 polymorphism with regular modest alcohol intake in ethnic Asians.

## 5. Limitations

This study had several limitations. First, this study did not involve the effects of a therapeutic intervention using aldehyde dehydrogenase 2; therefore, we were not able to demonstrate the concept that the observed LA substrate formation can be restored by ALDH2 supplements in regular drinkers carrying ALDH2 polymorphism. To this end, future interventional study design may be helpful to address this issue. Second, the generalizability of the observed associations of impaired LA mechanical booster pump function with binge alcohol use and incident AF in variant ALDH2 regular drinkers were limited to ethnic Asians and not tested in subjects of different races. These limitations may need to be clarified by further research.

## 6. Conclusions

Habitual alcohol consumption, although modest, impaired LA electromechanical parameters in an ethnic Asian cohort, leading to LA substrate formation with ALDH2 polymorphism. Mechanical LA substrates deteriorated with increasing alcohol consumption dose following a dose-response relationship and were more pronounced in subjects harboring ALDH2 variants. Except for the observed adverse effects on LA mechanical properties with regular alcohol consumption, binge-drinking behavior posed a substantial risk for impaired LA booster pump function. The application of novel LA mechanical parameters, particularly LA reservoir and booster functions, may serve as a useful clinical indicator for AF in Asian modest alcohol users.

## Figures and Tables

**Table 1:** Baseline demographic information, alcohol consumption patterns, and 4-HNE adduct levels across non-drinkers and habitual drinkers without and with the presence of ALDH2 polymorphism.

	Drinking Behavior			(Habitual Drinkers) (n = 193)		
	Non-Drinkers (n = 56)	Habitual Drinkers (n = 193)	*p*-Value	ALDH2 Polymorphism (−) (n = 135)	ALDH2 Polymorphism (+) (n = 58)	*p*-Value (ANOVA)
Demographics/anthropometrics						
Age, years	51.7 ± 10.0	51.4 ± 10.1	0.649	52.1 ± 10.4	49.5 ± 9.1	0.25
Female, %	20 (34.5)	29 (15.0)	0.002	20 (14.8)	9 (15.5)	0.007
Body height, cm	164.2 ± 9.1	167.7 ± 7.5	0.004	167.3 ± 7.5 *	168.6 ± 7.4 *	0.01
BMI, kg/m^2^	25.4 ± 3.5	25.9 ± 4.3	0.412	25.9 ± 4.5	25.9 ± 4.1	0.67
Waist circumference, cm	85.6 ± 10.1	88.1 ± 10.2	0.113	86.78 ± 10.23	88.69 ± 10.30	0.23
Systolic blood pressure, mmHg	129.7 ± 16.4	135.3 ± 18.4	0.048	137.1 ± 18.4 *	130.3 ± 17.5 ^#^	0.008
Diastolic blood pressure, mmHg	79.5 ± 11.3	82.8 ± 11.3	0.052	84.0 ± 11.2 *	80.4 ± 11.2	0.019
Pulse rate, beats/min	73.1 ± 12.9	72.7 ± 10.8	0.78	72.7 ± 10.6	72.5 ± 11.6	0.95
CHARGE-AF score	10.5 ± 1.1	10.8 ± 1.2	0.048	10.9 ± 1.1 *	10.5 ± 1.2	0.013
Alcohol consumption information						
Binge drinking, %	2 (3.6)	60 (31.1)	<0.001	43 (31.9)	17 (29.3)	<0.001
Daily alcohol intake, g/day ‡	0 [0~0]	14.1 [7.6~28.6]	<0.001	14.3 [8.3~28.6] *	12.3 [6.3~30.7] *	<0.001
Daily drinks, per day	0 [0~0]	1.2 [0.6~2.4]	<0.001	1.2 [0.7~2.4] *	1.0 [0.5~2.6] *	<0.001
Alcohol use duration, years	0 [0~0]	16.0 [5.0~30.0]	<0.001	20.0 [5.0~30.0] *	15.0 [5.0~30.0] *	<0.001
4-HNE level, μg/mL	34.2 ± 7.7	38.6 ± 9.5	0.002	36.6 ± 7.1 *	43.3 ± 12.5 *^,#^	<0.001
Medical history						
Smoking status			<0.001			<0.001
Non-smoker, %	43 (76.8)	74 (38.3)		48 (35.6)	26 (44.8)	
Active smoker, %	6 (10.7)	89 (46.1)		69 (51.1)	20 (34.5)	
Prior smoker, %	7 (12.5)	30 (15.5)		18 (13.3)	12 (20.7)	
Hypertension, %	10 (17.9)	89 (46.1)	<0.001	71 (52.6)	18 (31.0)	<0.001
Diabetes, %	11 (19.6)	48 (24.9)	0.42	29 (21.5)	19 (32.8)	0.17
Hyperlipidemia treatment, %	1 (1.8)	20 (10.4)	0.042	15 (11.1)	5 (8.6)	0.11
CAD, %	0 (0.0)	17 (8.8)	0.021	10 (7.4)	7 (12.1)	0.035
CVD, %	0 (0.0)	21 (10.9)	0.009	13 (9.6)	8 (13.8)	0.023
Biochemical data, %						
Hemoglobin, g/dL	14.26 ± 1.92	14.47 ± 1.45	0.39	14.45 ± 1.43	14.51 ± 1.50	0.66
Fasting sugar, mg/dL	107.2 ± 29.5	109.1 ± 32.2	0.69	105.6 ± 19.8	104.1 ± 23.7	0.88
Total cholesterol, mg/dL	195.2 ± 29.7	201.0 ± 37.7	0.30	205.2 ± 39.6	191.2 ± 31.2 ^#^	0.03
HDL-c, mg/dL	53.4 ± 14.3	53.0 ± 15.8	0.86	54.0 ± 15.3	50.9 ± 16.8	0.47
eGFR, mL/min/1.73m^2^	87.0 ± 18.3	93.4 ± 22.3	0.05	92.13 ± 21.32	95.11 ± 22.07	0.11

‡ Data are presented as median (IQR) with Kruskal-Wallis test results, including paired comparisons. * *p* < 0.05 vs. non-drinkers; ^#^ *p* < 0.05 vs. habitual drinkers with ALDH2 polymorphism (‒) using the ANOVA method.

**Table 2:** Comparisons of cardiac structure and novel LA mechanical parameters across non-drinkers and habitual drinkers without and with the presence of ALDH2 polymorphism.

	Non-Drinkers (n = 56)	Habitual Drinkers (n = 193)	*p*-Value	(Habitual Drinkers) ALDH2 Polymorphism (−) (n = 135)	(Habitual Drinkers) ALDH2 Polymorphism (+) (n = 58)	*p*-Value
Cardiac structure and function						
Septal wall thickness, cm	8.8 ± 1.3	9.3 ± 1.3	0.013	9.3 ± 1.3	9.4 ± 1.3	0.039
Posterior wall thickness, cm	8.9 ± 1.2	9.3 ± 1.3	0.027	9.2 ± 1.2	9.4 ± 1.5 *	0.048
Internal diameter (diastolic), cm	46.1 ± 4.2	47.0 ± 4.0	0.18	46.8 ± 4.2	47.4 ± 3.5	0.25
Mitral inflow E/A	1.16 ± 0.31	1.18 ± 0.47	0.75	1.16 ± 0.48	1.23 ± 0.46	0.58
LV mass index, kg/m^2^	71.8 ± 15.0	76.1 ± 16.0	0.074	75.6 ± 16.4	77.4 ± 15.2	0.16
Myocardial e’ (average), cm/sec	8.6 ± 2.1	8.3 ± 2.2	0.31	8.2 ± 2.2	8.4 ± 2.1	0.51
IVRT, ms	93.7 ± 20.8	88.8 ± 16.6	0.068	89.7 ± 17.8	86.8 ± 13.2	0.11
LV E/e’	8.0 ± 1.7	8.4 ± 2.5	0.29	8.3 ± 2.3	8.7 ± 3.0	0.29
LA electromechanical parameters						
PR interval, ms	152.9 ± 19.7	167.2 ± 23.9	<0.001	164.0 ± 23.8 *	174.4 ± 22.9 *^,#^	<0.001
LA volume (max), mL	36.8 ± 12.1	40.5 ± 13.9	0.07	38.7 ± 15.0	44.2 ± 13.2 *^,#^	0.007
LA emptying fraction (LAEF), %	43.6 ± 7.8	44.2 ± 8.2	0.66	44.8 ± 8.3	42.6 ± 7.6	0.19
LA volume index, mL/m^2^	19.4 ± 6.3	20.4 ± 6.4	0.30	19.7 ± 6.7	22.1 ± 5.5	0.036
PALS, %	35.1 ± 6.0	30.9 ± 6.2	<0.001	32.0 ± 6.1 *	28.2 ± 5.5 *^,#^	<0.001
LA reservoir function, 1/s	1.59 ± 0.30	1.33 ± 0.27	<0.001	1.38 ± 0.25 *	1.22 ± 0.28 *^,#^	<0.001
LA conduit function, 1/s	1.58 ± 0.51	1.38 ± 0.54	0.01	1.48 ± 0.54	1.13 ± 0.46 *^,#^	<0.001
LA booster pump function, 1/s	2.13 ± 0.50	1.88 ± 0.58	0.003	1.98 ± 0.55	1.62 ± 0.57 *^,#^	<0.001

* *p* remained <0.05 vs. non-drinker; ^#^ *p* remained <0.05 vs. habitual drinkers with ALDH2 polymorphism (−) using ANOVA method.

**Table 3:** Adjusted regression models that were used for examining the cardiac structure and novel LA mechanical parameters across non-drinkers and habitual drinkers without and with the presence of ALDH2 polymorphism.

	Non-Drinkers (n = 56)	(Habitual Drinkers) ALDH2 Polymorphism (−) (n = 135)	(Habitual Drinkers) ALDH2 Polymorphism (+) (n = 58)
Cardiac structure and function			
Septal wall thickness, cm	(Reference)	0.22 (−0.19, 0.63)	0.37 (−0.08, 0.82)
Posterior wall thickness, cm	―	0.01 (−0.39, 0.42)	0.29 (−0.16, 0.74)
Internal diameter (diastolic), cm	―	−0.33 (−1.59, 0.94)	0.39 (−1.01, 1.79)
Mitral inflow E/A	―	0.10 (−0.03, 0.24)	0.10 (−0.05, 0.24)
LV mass index, kg/m^2 †^	―	0.27 (−4.97, 5.52)	3.55 (−2.27, 9.36)
Myocardial e’ (average), cm/sec	―	0.11 (−0.44, 0.65)	−0.15 (−0.75, 0.45)
IVRT, ms	―	−2.5 (−8.5, 3.6)	−5.3 (−12.0, 1.4)
LV E/e’	―	0.3 (−0.4, 1.1)	0.9 (0.1, 1.8)
LA electromechanical parameters			
PR interval, ms	(Reference)	8.5 (0.7, 16.2) *	21.2 (12.6, 29.8) *^,#^
LA volume (max), mL	―	2.5 (−1.7, 6.7)	7.5 (2.8, 12.2) *^,#^
LA emptying fraction (LAEF), %	―	0.97 (−1.88, 3.82)	−1.15 (−4.31, 2.00)
LA volume index, mL/m^2 †^	―	0.9 (−1.3, 3.0)	3.2 (0.8, 5.6) *
PALS, %	―	−2.2 (−4.2, −0.4) *	−6.3 (−8.5, −4.2) *^,#^
LA reservoir function, 1/s	―	−0.18 (−0.28, −0.09) *	−0.38 (−0.49, −0.28) *^,#^
LA conduit function, 1/s	―	−0.001 (−0.15, 0.15)	−0.45 (−0.62, −0.28) *^,#^
LA booster pump function, 1/s	―	−0.08 (−0.27, 0.11)	−0.49 (−0.70, −0.28) *^,#^

^†^ Model in which BMI was not included. Data are presented as original coefficient values using ordinal categorical variables (three groups considered as non-drinkers, habitual drinkers with ALDH2 polymorphism (−), and ALDH2 polymorphism (+)) with non-drinkers considered as the reference group. * *p* remained < 0.05 vs. non-drinker; ^#^ *p* remained < 0.05 vs. habitual drinkers with ALDH2 polymorphism (−) after an adjustment for clinical covariates as confounders.

**Table 4:** The association between regular alcohol consumption and the presence of ALDH2 polymorphism with AF.

Prevalent AF	Non-Drinkers (n = 56)	(Habitual Drinkers) ALDH2 Polymorphism (−) (n = 135)	(Habitual Drinkers) ALDH2 Polymorphism (+) (n = 58)
Number of AF events	1 (1.8%)	15 (11.1%)	9 (15.5%)
Univariate model, OR (standard error (SE))			
	(Reference)	6.9 (7.19), *p* = 0.065	10.1 (10.8), *p* = 0.031
Multivariate model, OR (standard error (SE))			
Model adjusted for CHARGE-AF	(Reference)	5.6 (6.11), *p* = 0.11	12.7 (14.3), *p* = 0.024

## Data Availability

Because of the sensitive nature of the data collected for this study, requests to access the dataset from qualified researchers trained in human subject confidentiality protocols may be sent to “MacKay Memorial Hospital” Institutional Data Access/Ethics Committee for researchers (Institutional Review Board Contact information: MacKay Memorial Hospital. Address: No. 92, Sec. 2, Zhongshan N. Rd., Taipei City 10449, Taiwan. TEL: 02-25433535#3486~3488, E-mail: mmhirb82@gmail.com).

## Supplementary Materials

The following are available online at https://www.mdpi.com/article/10.3390/biom11111559/s1, Figure S1: Illustration on novel LA mechanical parameters using speckle-tracking based deformational algorithm, Table S1: The association of regular alcohol consumption duration (by decades) with LA mechanical parameters in current study.
