# Supplementary material for: Variant Aldehyde Dehydrogenase 2 (ALDH2*2) as a Risk Factor for Mechanical LA Substrate Formation and Atrial Fibrillation with Modest Alcohol Consumption in Ethnic Asians
Source: Biomolecules. 2021 Oct 21;11(11):1559. doi: 10.3390/biom11111559 (PMC8615757; doi:10.3390/biom11111559)
Supplement: Supplementary file 1 [file biomolecules-11-01559-s001.zip › biomolecules-1371609-supplementary.pdf]

# (Supplemental Materials)

**Figure S1. Illustration on novel LA mechanical parameters using speckle-tracking based deformational algorithm**

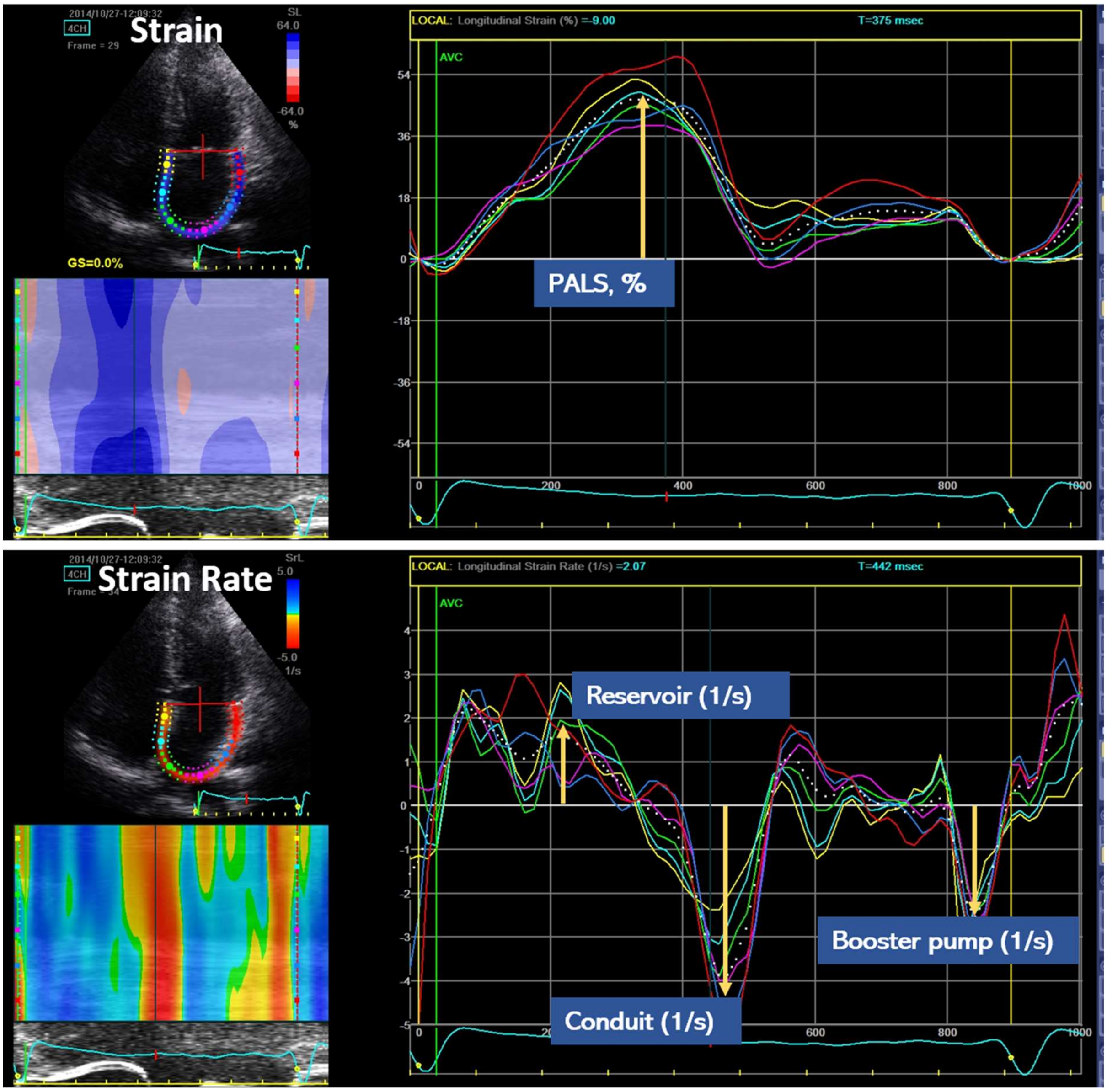

**Figure S2. The association of regular alcohol consumption duration (by decades) with LA mechanical parameters in current study** The number of participants was 126, 38, 48 and 55 for <10, 10-20, 20-30, and ≥30 years of regular alcohol use.

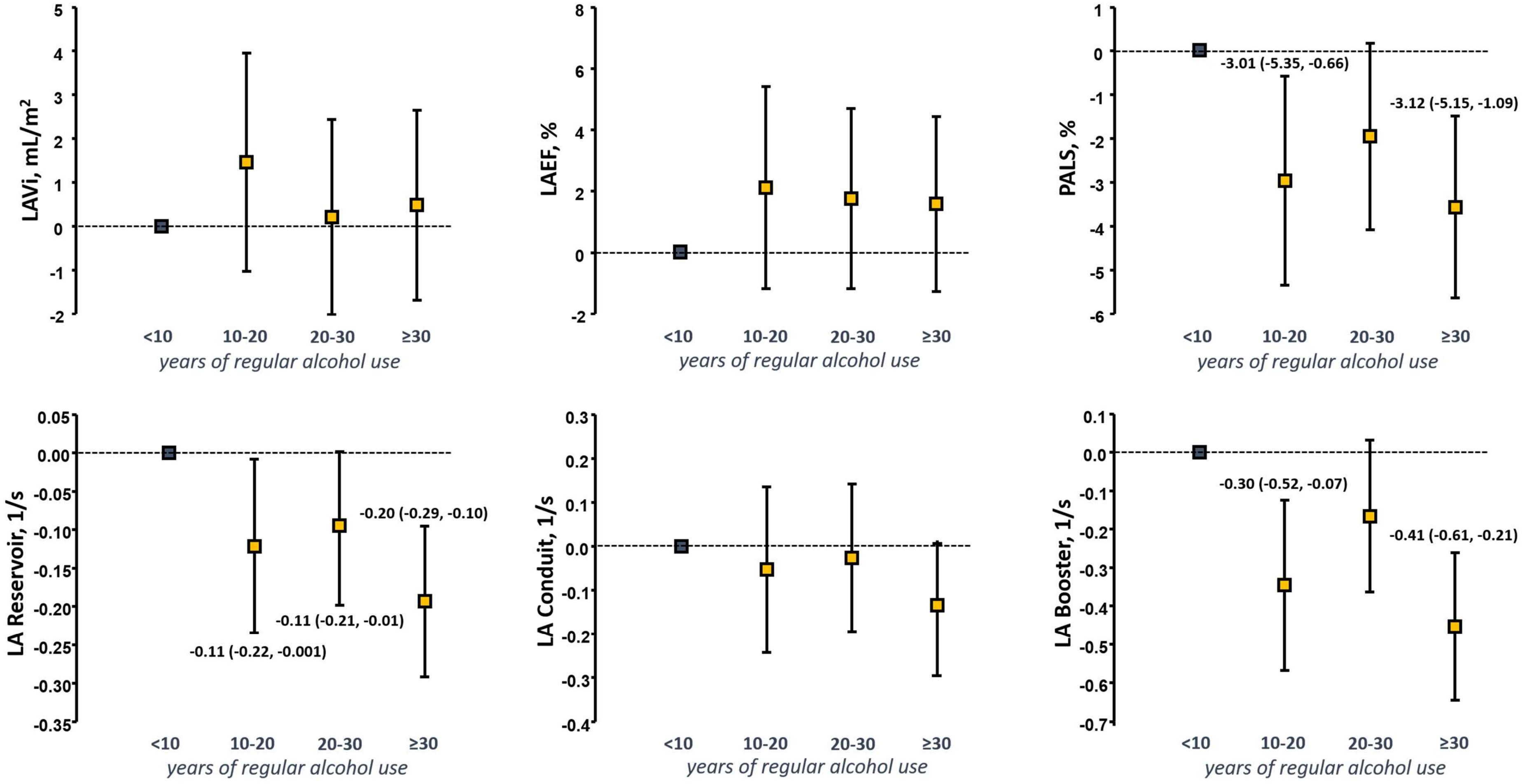

**Table S1.** Multi-variate linear regression models exploring predictors of mechanical and novel functional LA parameters in present study

| | LAVi, mL/$m^2$ | | LAEF, % | | PALS, % | |
|---|---|---|---|---|---|---|
| **Clinical Variables and ALDH2 Genotype** | Coefficient | 95% CI | Coefficient | 95% CI | Coefficient | 95% CI |
| **Age, +10 years** | 2.55 | 0.68 to 4.42* | 0.78 | -1.71 to 3.28 | -2.74 | -4.45 to -1.03* |
| **Female (Male as reference)** | 2.61 | 0.35 to 4.86* | -1.65 | -4.66 to 1.36 | -1.61 | -3.66 to 0.44 |
| **BMI, +5 kg/$m^2$** | 1.80 | 0.77 to 2.84* | -1.06 | -2.44 to 0.32 | -1.95 | -2.89 to -1.00* |
| **Daily alcohol intake, +10 gm/day** | 0.70 | 0.3 to 1.04* | -0.35 | -0.85 to 0.14 | -0.82 | -1.15 to -0.48* |
| **Hypertension** | 1.92 | -0.01 to 3.85 | -2.36 | -4.93 to 0.22 | -1.39 | -3.15 to 0.38 |
| **Diabetes** | -2.29 | -4.36 to -0.21* | -2.02 | -4.78 to 0.75 | --0.14 | -2.04 to 1.76 |
| **Hyperlipidemia treatment, %** | 1.00 | -2.00 to 4.00 | 2.39 | -1.62 to 6.40 | 0.32 | -2.43 to 3.08 |
| **CAD** | 5.81 | 2.64 to 8.99* | -4.83 | -9.06 to -0.59* | -2.85 | -5.75 to -0.08* |
| **Active Smoking** | 0.03 | -1.76 to 1.82 | 1.45 | -0.94 to 3.84 | -2.86 | -4.50 to -1.22* |
| **eGFR, +10 mL/min/1.73$m^2$** | 0.08 | -0.31 to 0.48 | 0.31 | -0.23 to 0.84 | -0.21 | -0.57 to 0.16 |
| **ALDH2 polymorphism ([-] as reference)** | 1.87 | 0.11 to 3.63* | -1.26 | -3.61 to 1.09 | -3.39 | -5.00 to -1.78* |

| | LA Reservoir, 1/s | | LA Conduit, 1/s | | LA Booster, 1/s | |
|---|---|---|---|---|---|---|
| **Clinical Variables and ALDH2 Genotype** | Coefficient | 95% CI | Coefficient | 95% CI | Coefficient | 95% CI |
| **Age, +10 years** | -0.06 | -0.14 to 0.02 | -0.43 | -0.56 to -0.31* | 0.03 | -0.13 to 0.18 |
| **Female (Male as reference) BMI,** | -0.06 | -0.16 to 0.03 | -0.05 | -0.20 to 0.10 | -0.28 | -0.47 to -0.09* |
| **+5 kg/$m^2$** | -0.07 | -0.12 to -0.03* | -0.18 | -0.25 to -0.11* | -0.11 | -0.20 to -0.02* |
| **Daily alcohol intake, +10 gm/day** | -0.04 | -0.06 to -0.02* | -0.06 | -0.09 to -0.03* | -0.03 | -0.07 to 0.003 |
| **Hypertension** | -0.08 | -0.16 to 0.002 | -0.08 | -0.21 to 0.05 | -0.04 | -0.21 to 0.12 |
| **Diabetes** | -0.001 | -0.09 to 0.08 | -0.006 | -0.14 to 0.13 | 0.07 | -0.10 to 0.25 |
| **Hyperlipidemia treatment, %** | -0.04 | -0.16 to 0.09 | -0.13 | -0.33 to 0.07 | 0.008 | -0.25 to 0.26 |
| **CAD** | 0.05 | -0.10 to 0.20 | 0.09 | -0.14 to 0.32 | -0.09 | -0.39 to 0.20 |
| **Active Smoking** | -0.03 | -0.11 to 0.05 | -0.09 | -0.21 to 0.03 | -0.29 | -0.44 to -0.14* |

| | | | | | | |
|---|---|---|---|---|---|---|
| **eGFR, +10 mL/min/1.73m²** | -0.01 | -0.03 to 0.01 | -0.01 | -0.04 to 0.01 | 0.004 | -0.03 to 0.04 |
| **ALDH2 polymorphism ([-] as reference)** | -0.10 | -0.17 to -0.02* | -0.34 | -0.46 to -0.22* | -0.29 | -0.44 to -0.14* |

* Denotes $p < 0.05$ for each variable in models. All mechanical and functional LA parameters were expressed as absolute values |x| for statistical convenience. Age, body mass index (BMI), and renal function in terms of eGFR were treated as continuous variables, whereas gender, medical histories of hypertension, diabetes, coronary artery disease, medications used for hyperlipidemia, and active smoking status (in contrast to non-smokers plus prior smokers) were treated as binary variables.
